# A Rare Case of Exercise-Induced Transient Global Amnesia as an Exclusive Presenting Symptom of Hippocampus Infarct

**DOI:** 10.7759/cureus.53866

**Published:** 2024-02-08

**Authors:** Roger F Tonna, Azin Azma

**Affiliations:** 1 Internal Medicine, Mountainview Hospital, Las Vegas, USA; 2 Neurology, Mountainview Hospital, Las Vegas, USA

**Keywords:** anterograde amnesia, memory loss, hippocampus, mri, diffusion-weighted image (dwi), cognitive decline, sports medicine, amnesia, transient ischemic attacks, transient global amnesia

## Abstract

Transient global amnesia (TGA) is a rare condition characterized by a temporary loss of the ability to form new memories. Retrograde episodic memory loss may also occur but to a lesser extent. Although TGA is generally benign, its sudden onset and similarity to more dangerous conditions like transient ischemic attack (TIA) or cerebral vascular accident (CVA) can be concerning. We present the case of a 70-year-old female who experienced confusion and general memory loss after a vigorous workout on her stationary exercise bike. After displaying considerable amnestic symptoms, she was admitted to the hospital for further medical attention and underwent a magnetic resonance imaging (MRI) that concluded a TGA diagnosis. This case report aims to investigate the prognosis associated with risk factors and refine the diagnostic criteria of TGA. We explore whether TGA caused by exercise, leading to unilateral or bilateral hippocampal lesions, is linked to cognitive decline. It is not yet clear if the development of TGA with unilateral infarct or bilateral hippocampal lesions results in different clinical presentations or varying prognoses. Further research is needed to determine the long-term risks of cognitive decline associated with resulting infarcts and clinical presentations.

## Introduction

Transient global amnesia (TGA) is a temporary loss of anterograde memory with a less prominent loss in retrograde episodic memory, which resolves within 24 hours or less. Despite being disoriented or confused about their surroundings, patients remain alert and communicative and display ordinary cognitive reasoning [1]. This syndrome has an acute onset that usually occurs in middle-aged and older individuals. It is a rare condition that occurs at a rate of 5.2-10/100,000 per year in the general population and 23.5-32/100,000 in individuals over 50 years old [2]. Common triggers of TGA include strenuous physical activity, sexual intercourse, head trauma, medical procedures, and others. Although considered a benign syndrome, when diagnosing TGA, a differential diagnosis must include cerebral vascular accident (CVA), transient ischemic attack (TIA), transient epileptic amnesia (TEA), hypoglycemia, and dissociative fugue [3]. In this case, we present a 70-year-old female patient who experienced confusion and isolated memory loss while exercising on the morning of admission. The patient had no significant medical or psychiatric history before this event. The patient's husband reported that she is health-conscious and exercises six days a week. Computerized tomography (CT) without contrast of the head showed no apparent causes, and a physical exam showed no focal neurological defects. Neurology was consulted, and brain magnetic resonance imaging (MRI) revealed a right hippocampal infarct. We present this case to highlight one of the causes contributing to TGA, emphasize the significance of effective clinical management, and underscore the distinct prognoses associated with different etiologies.

## Case presentation

Our patient was a healthy 70-year-old female who presented with the chief complaint of confusion and general memory loss. On the morning of admission, the patient remembered waking up around 7:00 AM and getting dressed to work out on her high-tech stationary bike at around 7:30 AM. She had recalled choosing a strength training workout on her stationary bike with handheld weights. She did not recall any events after starting her workout. Her husband stated that the exercise seemed strenuous, and he could hear her breathing hard from another room during the workout. He also mentioned that the last time she appeared normal was around 9:00 AM. After the workout, the patient told her husband she was confused and having trouble with her memory and wanted to go to the emergency room.

The patient stated that this had never happened before and had never had a memory lapse associated with exercising. The patient exercised six days a week and has been doing so her entire adult life. She had no history of migraines, seizures, or previous trauma or falls. At that time, the patient was not on any prescribed medications, and she denied the use of any tobacco products, alcohol consumption, or illicit drug use. She also denied any experience of having chest pain, shortness of breath, fevers, chills, nausea, vomiting, or weakness.

While in the emergency department, a rapid initial assessment was performed, and a code stroke was activated. The patient's vitals were stable. Given no risk factors for stroke and no other focal deficits, tenecteplase (TNK) was not recommended at that time. Neurology was consulted for further workup.

A thorough neurological examination was performed during the physical examination, and the patient demonstrated no focal deficits. Upon entering the room, the patient was awake and alert. The Glasgow Coma Scale was 14, as the patient stated she felt confused. Cranial nerves II through XII were intact; all four extremities demonstrated normal sensation and motor strength. The National Institutes of Health Stroke Scale (NIHSS) and Cincinnati Prehospital Stroke Scale were within normal limits. The patient was confused during the interview and occasionally repeated the same questions. When asked about the date and who the current president was, she could not recall. At the end of the interview, the patient could also not repeat what was said during the discussion nor recall the various brain imaging plans described to her.

The bloodwork obtained, which included a complete blood count, comprehensive metabolic panel, thyroid stimulating hormone, and urine drug screening, was unremarkable. A lipid panel showed mild hypercholesterolemia. An electrocardiography (ECG) showed sinus bradycardia; otherwise, it was unremarkable. A cranial CT scan without contrast showed no intracranial hemorrhage, mass, or evidence of acute ischemia. CT angiogram of the head with CT perfusion showed no large vessel occlusion, no ischemic penumbra, no acute hemorrhage, and no dural venous sinus thrombosis. CT angiogram of the neck showed 50% stenosis at the origin of the right common carotid artery and just distal to the origin of the left subclavian artery but patent cervical carotid and vertebral arteries without hemodynamically significant stenosis.

**Figure 1:**
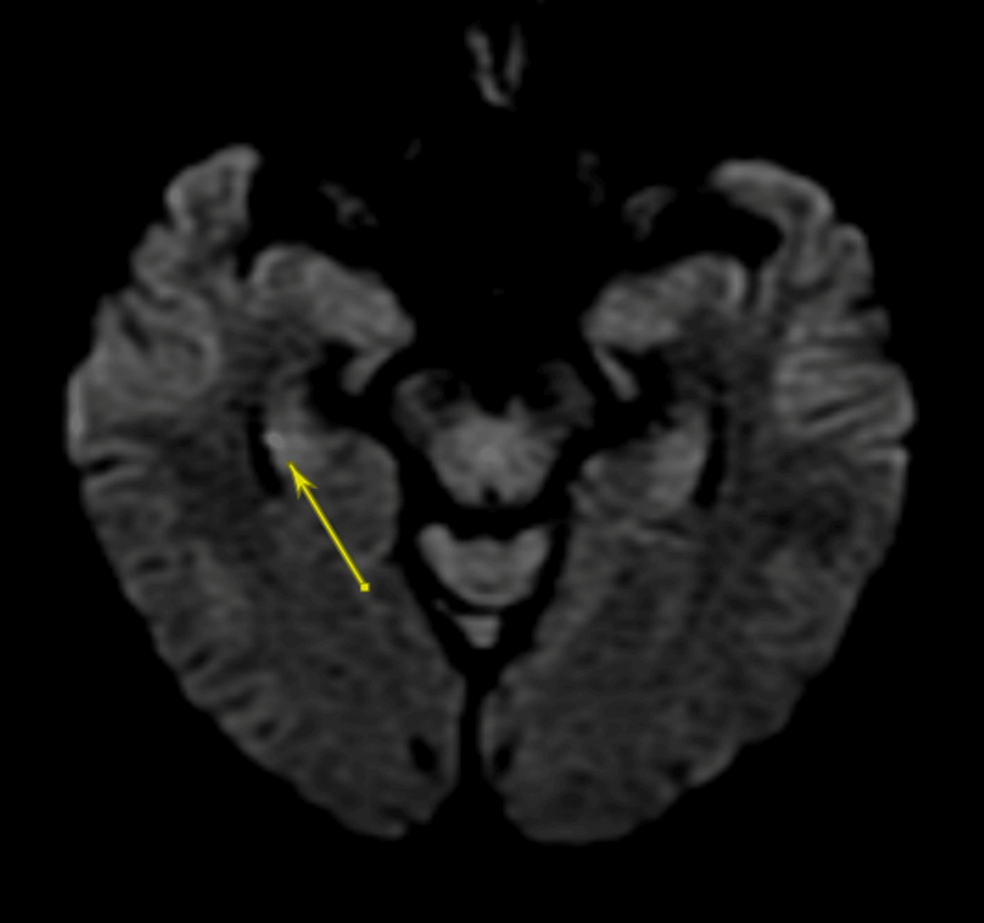
Axial view magnetic resonance diffusion-weighted imaging (DWI) showing a right 2 mm acute infarct of the midportion of the hippocampus

**Figure 2:**
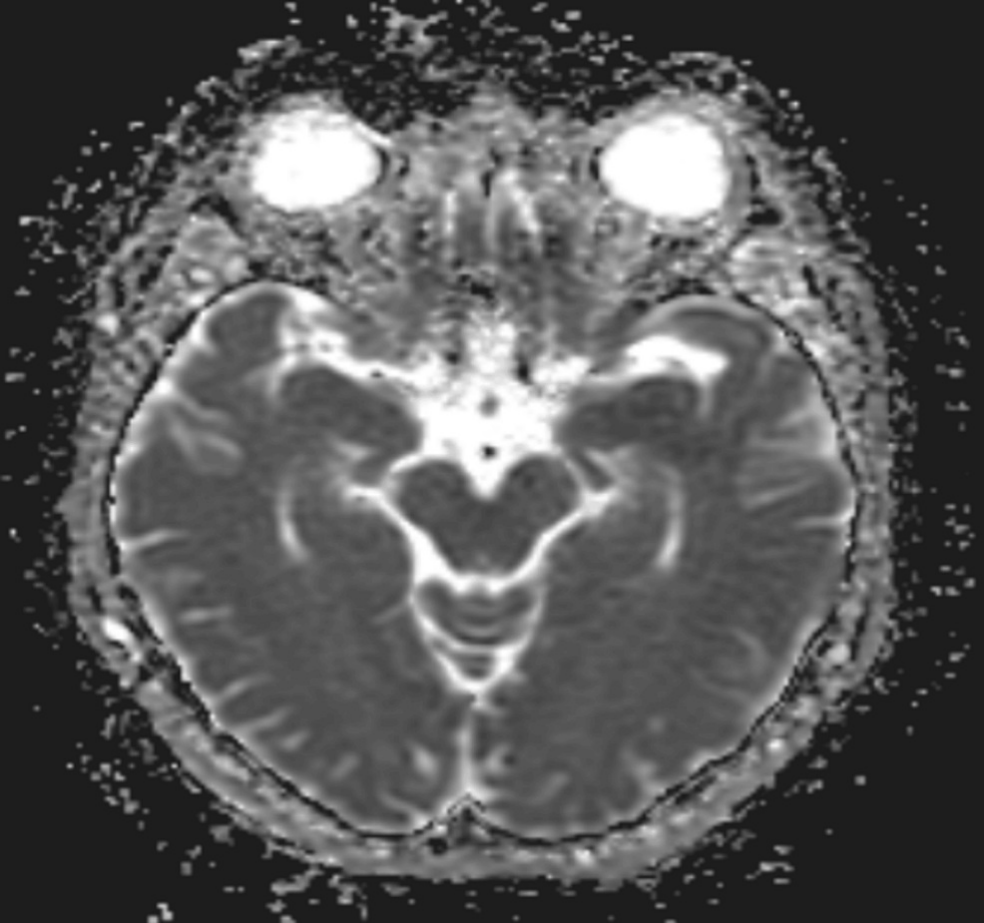
Apparent diffusion coefficient (ADC) MRI

**Figure 3:**
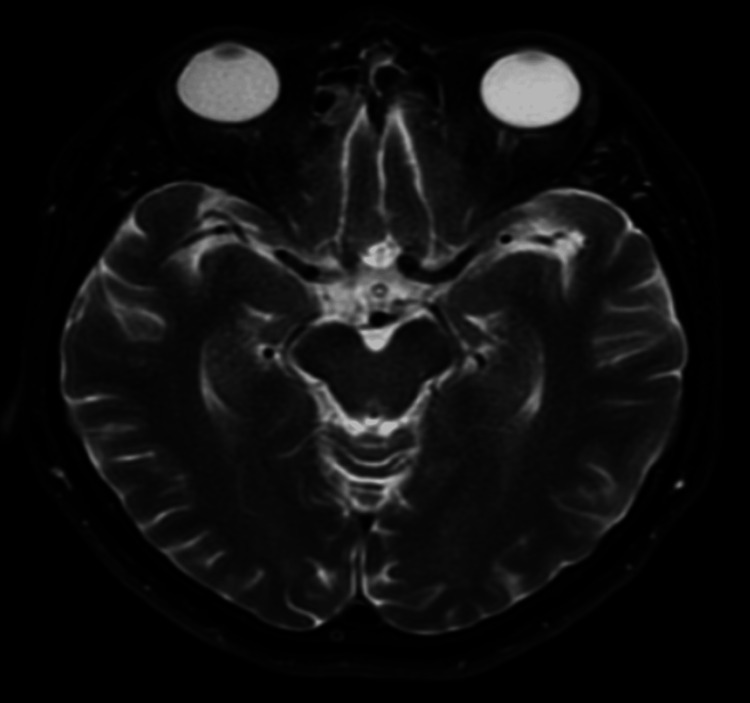
Axial view MRI T2-weighted sequence

A T1-weighted, T2-weighted, and diffusion-weighted MRI showed a 2 mm focus of restricted diffusion with minimal corresponding apparent diffusion coefficient (ADC) hypointensity of the midportion of the right hippocampus (arrow in Figure 1, Figure 2). No mass effect was observed due to its small size, and no hemorrhagic transformation occurred. No corresponding T2-weighted fluid-attenuated inversion recovery (FLAIR) hyperintensity was seen (Figure 3). An electroencephalogram (EEG) was performed and was unremarkable. An echocardiogram with bubble study showed normal left ventricular systolic function but noted a diastolic dysfunction. Her left ventricular ejection fraction ranged between 60-65%.

Later in the admission, her memory improved, and by the following morning, all of her amnestic symptoms had resolved. The neurologist diagnosed the patient with TGA using Hodges & Warlow’s criteria [1], as shown in Table 1. Upon discharge, she was started on dual antiplatelet therapy of daily aspirin 81 mg and clopidogrel 75 mg for three weeks and then transitioned to only 81 mg daily of aspirin. She was also placed on a statin. We followed up with the patient six months after her TGA episode, and there were no recurring episodes. She has returned to exercising six days a week.

**Table 1:** Hodges & Warlow criteria for transient global amnesia (TGA)

Diagnostic criteria for definite TGA [1]
Attacks must be witnessed, and information must be available from a capable observer who was present for most of the attack.
There must be clear-cut anterograde amnesia during the attack.
Clouding of consciousness and loss of personal identity must be absent, and the cognitive impairment limited to amnesia (that is, no aphasia, apraxia, etc).
There should be no accompanying focal neurological symptoms during the attack or significant neurological signs afterward.
Epileptic features must be absent.
Attacks must be resolved within 24 hours.
Patients with recent head injury or active epilepsy (that is, remaining on medication or one seizure in the past two years) are excluded.

## Discussion

When diagnosing TGA, excluding differential diagnoses with similar symptoms is crucial. Other diagnoses that have a similar presentation as TGA include TIA, acute ischemic stroke, seizure disorders, infectious or inflammatory conditions, and encephalopathies affecting the hippocampus. All of these conditions can affect the hippocampus and mimic the symptoms of TGA [4]. TGA is a diagnosis of exclusion. Thus, a workup should include a blood panel, a physical examination, and an MRI of the brain to support clinical suspicion of TGA. An MRI with DWI has the highest specificity to distinguish the various types of distinct lesion patterns in pathologies affecting the hippocampus [4].

Timing of the MRI is an important consideration, as during the acute phase of the disease, lesions may not become visible up to 48 hours post-initial symptoms. Previous observational studies have noted that about 6% of patients displayed punctate hippocampal DWI changes in the acute phase, but 70-80% did so after 24-48 hours [4,5]. Classic findings seen on DWI are hyperintense lesions with a diameter of approximately 1-3 mm in the lateral aspect of the hippocampus [6]. These findings highlight the use of serial MRIs as an essential diagnostic modality.

The exact etiology of TGA is still unknown, but various mechanisms have been suggested, including hypoxemia secondary to venous congestion, cortical spreading depression, hypoperfusion-induced ischemia, and arterial infarcts. The leading pathophysiologic mechanism proposed is abnormal venous drainage in the medial temporal lobes, which causes reactive arterial vasoconstriction, venous hypertension, and hypoperfusion to the area [7]. Known precipitating factors that have caused this congestion include the increased intrathoracic pressure after a Valsalva maneuver preventing venous return to the superior vena cava and reactive arterial vasoconstriction during hyperventilation. Vasoconstriction due to hyperventilation during emotional arousal can cause cerebral hypoperfusion and impaired hippocampal activity [7]. Thus, hypoperfusion caused by venous congestion or vasoconstriction due to hyperventilation could cause TGA. In another case report, the hypothesis that temporal hypoxic-ischemic dysfunction induced by hemodynamic factors was the cause of TGA was supported using MR and SPECT images, correlating with the particular anatomy of the affected hippocampus [8]. During increased cellular metabolic stress (i.e., hypoxemia and ischemia), the lateral portion of the hippocampus is most susceptible to damage, specifically the cornu ammonis 1 (CA1) neurons, as they are supplied by only one sizeable ventral artery. CA1 neurons are involved in the process of memory consolidation, and this can account for why memory structures are first affected [6]. It can be assumed that prolonged ischemia to the hippocampi can cause atrophy, increase cognitive decline, and potentially develop Alzheimer's disease.

Very few cases have been explained using the proposed pathophysiology as the cause of TGA, and the majority of those cases were exertion-induced TGA from a case of a 52-year-old man with headaches triggered by sexual intercourse to a 21-year-old collegiate pitcher with type 1 diabetes [9,10]. Coincident health conditions have been recognized as risk factors for developing TGA in all those cases. Our patient had no significant comorbidities and lived a very active lifestyle, performing aerobic exercise six days a week. In this case, the onset of vigorous exercise likely caused an increase in retrograde flow. Venous congestion was likely exacerbated by a relatively compromised right carotid artery stenosis. This resulted in arterial insufficiency and subsequent infarct of the right midportion of the hippocampus, leading to the development of TGA.

The patient had an echocardiography that showed she had an ejection fraction of 60-65% but with corresponding diastolic dysfunction. The patient has been training for many years, and her echocardiography suggests signs of exercise-induced cardiac remodeling (EICR). Several studies have shown that EICR is a continuous process responsive to a sustained or repetitive exercise stimulus [11]. These studies demonstrated that left ventricular hypertrophy can be seen within three months of training and begins to occur with as little as three to four hours of exercise a week [11]. TGA has occurred in other cases after maximal graded exercise testing [12]. Even though amnesia is reasonably recognizable, it is essential to rule out other exercise-induced severe conditions such as ischemic stroke, TIA, or diffuse cerebral hypoperfusion [12,13].

On the other hand, it is crucial to recognize that exercise testing can trigger a TGA episode in specific prone individuals and should not be interpreted as a disorder that initially requires invasive and potentially harmful evaluations (i.e., invasive catheterization) [12]. Alternatively, exploring non-invasive options should be considered initially, and if necessary, medical therapy should be started [11]. This approach can be more effective and less risky in some cases, so it is worth considering as an initial step.

What makes our case unique is the severity of the patient's presenting symptoms compared to the findings seen on imaging and the lack of medical history. Usually, unilateral hippocampal involvement in ischemic events may cause no or very subtle memory impairments [4]. Our patient had a right 2 mm infarct and developed pronounced transient memory disturbance, generally seen in bilateral hippocampal infarctions. It is hypothesized that during global hypoxia, bilateral hippocampal ischemia causes the associated loss of declarative memory seen in TGA [9].

TGA is a benign condition which generally resolves within 24 hours. Rarely will there be complications during the initial presentation of symptoms, but the long-term risk of developing dementia is guarded. In a control cohort study, 181 TGA subjects and 543 non-TGA controls were followed up after eight years [14]. Of the 181 TGA subjects, 14 had developed dementia, with yearly incidence rates of 20.14 per 1,000 persons. When accounting for age, gender, and comorbidities, the calculated adjusted hazard ratio (HR) for dementia in TGA cohorts was 2.23 (95% CI [1.12-4.44], p = 0.023) versus with non-TGA cohorts [14]. In other comparative studies, 55 patients were followed up one year after having TGA, and 18 of the 55 patients (32.7%) developed mild cognitive decline as per the amnestic mild cognitive impairment (MCI-a) criteria [15]. Many of these studies adjusted the HR for age, gender, and comorbidity, but little was mentioned in the prognosis of bilateral ischemia compared to unilateral ischemia. In this manner, more information is needed regarding the long-term risk of the various findings and etiologies associated with TGA.

## Conclusions

This case report aims to highlight the need for further research on the potential risks of developing cognitive decline when diagnosed with TGA. Current research has not concluded whether developing bilateral hippocampal lesions increases the risk of developing cognitive decline than a unilateral infarct in associated exercise-induced TGA cases. Nor has it been established if the severity of the amnestic syndrome plays a role in the development of cognitive decline. 

This case serves as a reminder that even patients with healthy lifestyles or limited risk factors can develop exercise-induced TGA and require treatment with frequent outpatient follow-ups to prevent the long-term consequences of cognitive decline. There is a need for further research into the clinical outcomes of patients who develop TGA, focusing on addressing the potential risks associated with the various causes. It is critical for the athlete's well-being that treatment and surveillance protocols be tailored to their specific needs. Recognizing the various outcomes of TGA, paired with the appropriate interventions, will ultimately improve the patient's long-term prognosis.
